# A Case of Early Disseminated Neurological Lyme Disease Followed by Atypical Cutaneous Manifestations

**DOI:** 10.1155/2017/6598043

**Published:** 2017-04-23

**Authors:** Vamsi Kantamaneni, Vikas Sunder, Mohammad Bilal, Scott Vargo

**Affiliations:** Department of Internal Medicine, Allegheny General Hospital, Allegheny Health Network, Pittsburgh, PA, USA

## Abstract

Lyme disease (LD) is a tick-borne illness caused by* Borrelia burgdorferi sensu stricto*. An 80-year-old female from Pennsylvania, USA, presented to an outside hospital with fever, confusion, lower extremity weakness, and stool incontinence. CT head and MRI spine were unremarkable. An infectious work-up including lumbar puncture was negative. She was transferred to our tertiary care hospital. Patient was noted to have mild unilateral right-sided facial droop and a diffuse macular rash throughout the body. She denied any outdoor activities, tick bites, or previous rash. Intravenous ceftriaxone was started for suspected LD. The patient's symptoms including facial droop resolved within 24 hours of antibiotic therapy. Polymerase chain reaction of the blood, IgM ELISA, and IgM Western blot testing for LD came back positive a few days after initiation of therapy. She was treated for a total of 21 days for neurological LD with complete symptom resolution. Not all patients have the classic “targetoid” EM rash on initial presentation, rash could develop after neurological manifestations, and prompt initiation of antibiotics without awaiting serology is paramount to making a quick and a full recovery. There should be a high index of suspicion for early disseminated LD, as presentations can be atypical.

## 1. Introduction

Lyme disease (LD) was originally identified in Lyme, Connecticut, when a cluster of patients presented with an apparent juvenile rheumatoid arthritis. Previously known as Lyme arthritis, the entity is now called LD because of its wide variety of clinical manifestations including cardiac, dermatological, and neurological findings [1]. LD is the most common tick-borne infection in the northern hemisphere. In North America, LD is predominantly caused by* Borrelia burgdorferi sensu stricto*. Since it was first recognized as a clinical entity in 1975, the number of cases in the United States has increased [2]. According to the Center for Disease Control (CDC), the actual number of annual cases is close to 300,000 [3]. Transmission occurs secondary to a tick bite containing a spirochete in a genetically susceptible host [1]. Prolonged attachment allows time for the spirochete to transmit from tick to the human body [4].

LD classically begins with an erythematous rash called erythema migrans (EM). The rash may have centrally located vesicles or necrotic areas [5]. Although EM lesions may occur anywhere on the body surface, common sites are the groin and axilla and, in children, the head and neck [5]. In children, the lesion can even occur periorbitally [6]. The rash is classically described as targetoid or bull's eye appearance, with an area of erythema surrounded by central clearing [7].

Hematogenous dissemination from the initial skin lesion is thought to cause secondary skin lesions and extracutaneous manifestations. The most common sign of early disseminated infection is multiple, often smaller EM lesions. The time duration at which hematogenous dissemination occurs remains unknown [8]. Approximately 4–8% of patients develop cardiac symptoms and 11% develop neurologic symptoms [1]. Arthritis is usually seen in late disease and occurs in 45–60% of untreated patients [1]. Patients typically present approximately six months after infection with joint pain and swelling which primarily involves the knees and hips [9].

We present a case of LD at our institution where the patient presented with nonspecific neurological symptoms followed by an atypical macular rash later in the clinical course.

## 2. Case Presentation

An 80-year-old Caucasian female, resident of the state of Pennsylvania, USA, with a past history of hypertension, right-sided thyroidectomy, and stroke without residual deficits presented to an outside hospital in the month of June 2015 with fever, confusion, headaches, bilateral lower extremity weakness, and an episode of stool incontinence. Basic lab work-up including total white count was within normal limits.

Computed tomography (CT) scan of the head did not show any acute abnormalities. Magnetic resonance imaging (MRI) of the thoracic and lumbar spine performed for concerns of spinal cord compression was unremarkable. An infectious work-up including chest X-ray and urine and blood cultures was negative. Lumbar puncture was performed and it did not show any lymphocytic pleocytosis or findings suggestive of an infectious process. Due to the complexity of her presentation, she was transferred to our tertiary care hospital after spending 3 days at the outlying facility.

**Figure 1:**
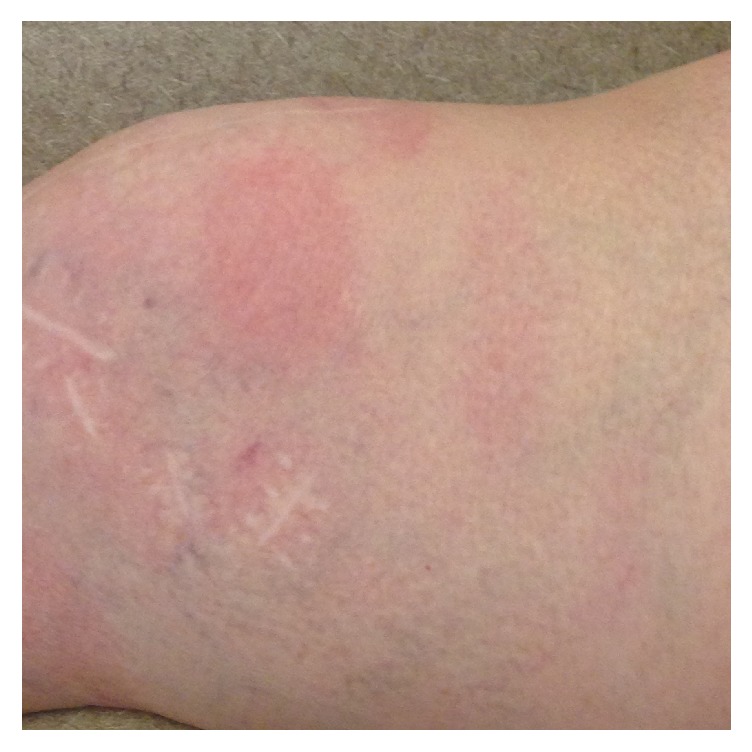
Macular rash over the right knee.

**Figure 2:**
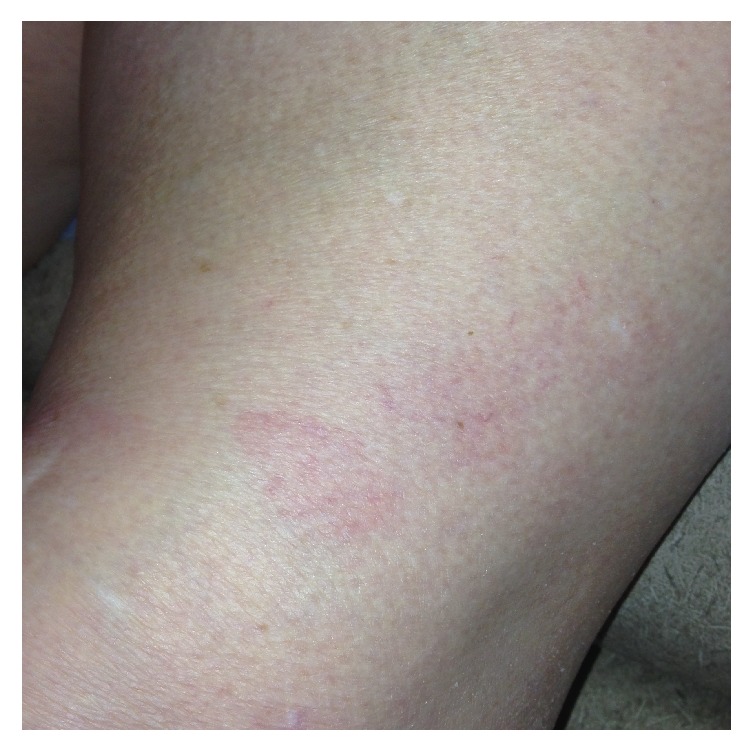
Macular rash over the left knee.

On presentation to our hospital, the patient continued to complain of severe headaches and was noted to have mild unilateral right-sided facial droop and a diffuse macular rash throughout the body (Figures 1 and 2). She denied any outdoor activities, recent tick bites, or noticing any previous rashes. There was no neck stiffness or photophobia. She was started on intravenous ceftriaxone for suspected LD as she comes from an endemic region and has an uncharacteristic rash with neurological manifestations. Unfortunately, cerebrospinal fluid testing for LD was not performed at the outside facility. The patient's headache, fever, lethargy, and her neurological manifestations including facial droop resolved within 24 hours of antibiotic therapy.

Due to high suspicion of early disseminated LD in our patient, Lyme serology was ordered. Testing included screening ELISA, which was positive for IgM antibody, confirmed by IgM positive Western blot test (IgM positive for 39, 41; IgG positive for 23 and 41). Western blot testing was negative for IgG as only two of the 10 bands came back positive, the required being five out of ten. Real-time polymerase chain reaction (PCR) of the blood sample tested positive for DNA of* Borrelia burgdorferi sensu stricto.* Of note, results of the Western blot were available a few days after the discharge of the patient from the hospital.

Our patient did not have the classic “targetoid” EM rash on initial presentation. Another unique feature was development of the rash after initial neurological manifestations. She was treated for a total of 21 days for neurological LD. The patient's rash and her neurological manifestations had completely resolved. At a 3-month follow-up, the patient reported no further episodes of headaches, rash, and fevers.

## 3. Discussion

LD is the most common tick-borne infectious disease in North America and in countries with temperate climates in Europe and Asia [10]. Early LD is a clinical diagnosis in endemic areas in patients presenting with EM rash, predominantly in the months of May through August. Serologic testing can be misleading because the false negative rate is as high as 60% in the first 2–4 weeks of infection [11].

The LD cases are concentrated in the Northeast and upper Midwest of USA, with 14 states accounting for over 96% of cases reported to CDC. Of the cases reported to CDC, it is most commonly seen among boys aged 5 to 9 years. LD patients are most likely to have illness onset in June, July, or August and less likely to have illness onset from December through March. As per CDC estimates from the years 2001 to 2015, in USA, on an annual basis, there are more than 300,000 cases of LD with the months of June and July being the highest with greater than 75,000 LD cases each.

The clinical manifestations of LD can be divided into three stages: early localized infection, early disseminated disease, and late infection [10]. Early localized infection of LD occurs 7–10 days after a tick bite and the most typical presentation is the characteristic EM rash. It is present in up to 80% of the patients during early localized stage. Early disseminated disease occurs weeks to months later; patients can have secondary skin lesions, lymphadenopathy, migratory joint and muscle pain, meningitis with cranial nerve involvement, and Lyme carditis. Late infection in the United States is characterized by arthritis as a common feature; neurologic manifestations such as a subtle encephalopathy or polyneuropathy can also occur [10, 12]. In Europe, cutaneous manifestations such as acrodermatitis chronica atrophicans are commonly reported [13].

Lyme carditis may present very early as a part of early disseminated disease. It can cause self-limited conduction abnormalities with varying degrees of atrioventricular block with 3rd-degree heart block being the most common. Rarely, it can cause fatal ventricular arrhythmias. The likely pathogenesis here is direct invasion of myocardial tissue and inflammatory reaction caused by* Borrelia burgdorferi*. It may manifest as pericarditis, myocarditis, and endocardial fibrosis [10].

Most patients with LD have normal blood counts like in our patient. The presence of leukopenia, thrombocytopenia, and high-grade fever should raise suspicion for anaplasmosis, while the presence of severe anemia alone should raise suspicion of babesiosis [11]. When there is a suspicion of either babesiosis or anaplasmosis, PCR test can be used for confirmation [11]. Mehrzad and Bravoco reported a unique case of pancytopenia in a patient with LD. Coinfection with ehrlichiosis and anaplasmosis was ruled out. This case illustrated that* Borrelia burgdorferi* has the potential to induce hemolytic anemia. Oxygen labile hemolysin enzymes are associated with certain strains of leptospirosis [14]. This gives credence to the finding of pancytopenia in the case reported by them.

Atypical presentation of LD can pose a diagnostic challenge. Early neurologic presentations typically include cranial neuritis, radiculitis, and meningitis. Horner syndrome is a rare manifestation of neurologic Lyme [11]. A case of pseudotumor cerebri was reported by Kan et al. In this patient, impaired CSF flow and raised intracranial pressure could have resulted as a consequence of direct infective or inflammatory process. There were around 12 such cases reported. However, the prognosis of pseudotumor cerebri when associated with LD appears to be good when treated appropriately [15].

Lyme meningitis is indistinguishable from viral aseptic meningitis. On lumbar puncture, the expected finding would be lymphocytic pleocytosis; however, our patient had neither an increase in CSF lymphocytes nor peripheral leukocytosis. He presented with nonspecific neurological symptoms like headaches, confusion, and unilateral facial droop and features suggestive of spinal cord compression such as bilateral lower extremity weakness and bowel incontinence. There was also an initial concern for cerebrovascular accident such as stroke but imaging did not provide any such evidence. The development of diffuse rash along with the neurological symptoms made us consider the possibility of LD.

Our patient did not have the classic “targetoid” EM rash on initial presentation. Another unique feature was development of the rash after initial neurological complaints. Given the symptomatology and patient being from an endemic area of the United States, there was a high suspicion of early disseminated LD. Lyme serology was ordered and the patient was started on intravenous ceftriaxone. The symptomatology immediately improved with appropriate antibiotic treatment. A few days after discharge of the patient from the hospital, the confirmatory Western blot test for LD came back positive.

Our case highlights the fact that the suspicion of LD should be very high in endemic areas. The lack of the classic bull's eye appearing rash and typical symptoms should not completely exclude the presence of LD. Serologic testing for antibodies is an adjunct to the clinical diagnosis and can neither establish nor exclude the diagnosis of LD. A positive or negative serologic test simply changes the probability that a patient has been infected with* Borrelia burgdorferi*. This has to be interpreted in clinical context. In suspected early disseminated LD, the treatment with intravenous antibiotics should be started immediately without waiting for serology as prompt initiation of antibiotics is paramount to making a quick and a full recovery. Our case is peculiar due to the atypical nature of the rash and occurrence of early disseminated neurological disease before the development of diffuse rash. There are a few such other atypical cases reported in the literature (Table 1).

## Figures and Tables

**Table 1:** Some of the atypical presentations of Lyme disease reported in the literature.

Age	Sex	Clinical presentation	Diagnostics	Treatment	Author
69	F	4-Day history of right eye pain, fever, fatigue, unequal pupils, and ptosis; diagnosed to be having *Horner syndrome* after a positive cocaine stimulation test. Skin exam showed an *atypical vesicopustular variant of erythema migrans*. After treatment, Horner syndrome resolved.	Initially Lyme antibody was negative; 4 weeks later, it turned positive. CSF culture positive for *Borrelia burgdorferi*	Intravenous (IV) ceftriaxone for 4 weeks	Morrison et al. [11]

25	F	1-Month history of *sudden onset hearing loss* along with fever, *vertigo*, nausea, and vomiting. 2 months prior to presentation, she had *unsteady gait* and several episodes of fever. 4 months later, she developed left sided facial palsy. 5 years ago, the patient recalled having a circular rash. After treatment, facial palsy improved with resolution of fevers and vertigo.	MRI of brain was negative. Serology for syphilis and HIV was negative, and Western blot for IgM/IgG was positive for Lyme	Ceftriaxone IV for 4 weeks	Peeters et al. [16]

30	M	Presented with headache, neck pain, dizziness, tenderness behind the ears, weight loss, and unsteady gait. After treatment, there was complete clinical resolution.	CT of head and MRI of head and neck were negative. IgG against VlsE C6 peptide of *B. burgdorferi* was positive	Ceftriaxone IV for 2 weeks	Winter et al. [17]

17	M	Developed fever, sore throat, cough, fever, myalgia, diarrhea, and lightheadedness. Serology for Lyme disease and anaplasmosis was negative. Chest X-ray showed cardiomegaly. EKG with *prolonged PR* interval. He was tachycardic and febrile. On arrival to tertiary care facility, he developed *ventricular fibrillation* leading to death. Autopsy revealed diffuse lymphocytic pancarditis.	Lumbar puncture (LP) revealed lymphocytic pleocytosis. ELISA and IgM Western blot were positive. Immunohistochemistry and real-time PCR were positive in myocardium, lung, and brain tissue		Yoon et al. [10]

46	M	Presented with fatigue, presyncope, and palpitations found to be bradycardic in 3rd-degree *atrioventricular (AV) block*. Transvenous pacemaker was placed. Later developed unilateral *Bell's palsy*. With treatment, his 3rd-degree AV block converted to normal sinus rhythm 3 days later.	EKG Serology was positive for Lyme Echocardiogram showed no abnormality, with ejection fraction of 65%	Ceftriaxone IV	Lee and Singla [18]

49	M	Presented with fevers, chills, fatigue, and unilateral lower extremity swelling. He had dark colored urine. Was found to be hypotensive and was given vancomycin and ampicillin-sulbactam. Lactic acid, aminotransferase, and alanine transaminase were elevated. Blood work further showed *anemia*, *leukopenia*,* and thrombocytopenia*, increased lactate dehydrogenase, and decreased haptoglobin.	Positive ELISA and IgM Western blot for Lyme. *Negative serology for Ehrlichiosis*,* Anaplasmosis*, and HIV. Positive for parvovirus B19 IgG	Doxycycline 100 mg	Mehrzad and Bravoco [14]

8	F	Presented with acute onset of headache and diplopia. She was found to be having left 6th cranial nerve palsy and bilateral papilledema. CT and MRI of head were normal. She was found to be having *pseudotumor cerebri* secondary to acute neuroborreliosis. After treatment, she had resolution of symptoms except for mild residual left 6th cranial nerve palsy without papilledema.	LP revealed elevated pressure with lymphocytic pleocytosis. Lyme ELISA positive, IgM 23, 37, 39, 41 positive, IgG 39, 41, 45, 58	Ceftriaxone IV for 4 weeks and acetazolamide	Kan et al. [15]
